# Supplementary material for: Classifying and visualizing medication use in the Adolescent Brain Cognitive Development (ABCD) Study
Source: medRxiv. 2025 Nov 20:2025.11.19.25340321. Preprint. [Version 1] doi: 10.1101/2025.11.19.25340321 (PMC12668116; doi:10.1101/2025.11.19.25340321)
Supplement: 1 [file NIHPP2025.11.19.25340321V1-supplement-1.pdf]

medRxiv preprint doi: https://doi.org/10.1101/2025.11.19.25340321; this version posted November 20, 2025. The copyright holder for this preprint (which was not certified by peer review) is the author/funder, who has granted medRxiv a license to display the preprint in perpetuity.
It is made available under a CC-BY 4.0 International license .

Table S1. Items on the Brief Problem Monitor - Internalizing Scale

1. I feel worthless or inferior
2. I am too fearful or anxious
3. I feel too guilty
4. I am self-conscious or easily embarrassed
5. I am unhappy, sad, or depressed
6. I worry a lot

Possible Responses were 0=Not True, 1=Somewhat True, 2=Very True

medRxiv preprint doi: https://doi.org/10.1101/2025.11.19.25340321; this version posted November 20, 2025. The copyright holder for this preprint (which was not certified by peer review) is the author/funder, who has granted medRxiv a license to display the preprint in perpetuity.

It is made available under a CC-BY 4.0 International license .

Table S2.

Estimated Use Category	Unique Medication Count
Cold/Flu/Allergy Products	553
Dermatologic - Systemic and Topical Acne Treatments	355
Systemic Antihistamine	340
Pain Treatment - Non-Opioid Analgesic	324
Contraceptive or Hormonal Regulation	234
Asthma / Obstructive Airway Disease	233
Dermatologic - Topical Corticosteroid	176
ADHD Medication - Methylphenidate Based (Stimulant)	164
Antidepressant - Non-SSRI	134
GI/Acid-Related Treatments	128
Systemic Antibiotic - Penicillins	124
Constipation Treatment and Laxative Agents	119
Miscellaneous	119
Antidepressant - SSRI	115
Dietary Supplement - Miscellaneous	114
Dietary Supplement - Extract	108
Antipsychotic - Typical and Atypical	101
Any Cardiovascular/Renal Medication	93
ADHD Medication - Amphetamine Based (Stimulant)	92
Hypnotic/Sleep Aid	90
Local Corticosteroids	90
Local Antihistamines - Nasal/Eye	83
Anxiolytic Medications - Benzodiazepines, Beta Blocker, Buspirone, or Sedating Antihistamine	80
Pain Treatment - Opioid	79
Gastrointestinal Support	75
Insulin - (Rapid/Intermediate/Long/Premixed)	75
Dermatologic - Systemic and Topical Antifungals	74
Systemic Corticosteroid	74
Opthalmic Agents	72
Mental Health - Mood Stabilizer	71
Migraine/Headache Treatment	71
ADHD Non-Stimulant - Alpha-2 Adrenergic Agonist	70
Dietary Supplement - B Vitamins (Biotin, Folic Acid, Vitamin B-12, Vitamin B-6)	70
Immunomodulators & Cytotoxic Agents	70
Vaccine	67
Antacid / Antidiarrheal / Nausea - Symptom Relief	65
Topical Antibiotic	62
Dental / Oral Health and Hygiene	55
Digestive Health - Probiotic Supplement	55
Cardiovascular Supplement - Omega-3 / Omega-6 Fatty Acids	53
Allergic or Atopic Disease Medications	52
Systemic Antibiotic - Cephalosporins	51
Thyroid Medications (Hypothyroidism and Hyperthyroidism)	50
Dietary Supplement - Magnesium	49
Broad Antiviral Use	48

medRxiv preprint doi: https://doi.org/10.1101/2025.11.19.25340321; this version posted November 20, 2025. The copyright holder for this preprint (which was not certified by peer review) is the author/funder, who has granted medRxiv a license to display the preprint in perpetuity.

It is made available under a CC-BY 4.0 International license .

Sex Hormone Therapy	48
Systemic Antibiotic - Other	44
Antidiabetic (Non-insulin) and Weight Management Medications	41
Other Dermatologic Treatments	36
Antiepileptic	35
Antiparasitic Medications	33
Dermatologic - Moisturizer/Barrier Agent/Itch Relief	33
Iron Supplement / Antianemic Agent	33
Urologic Medications	33
Antiemetic	30
Dietary Supplement - Vitamin D	30
Bone & Calcium Support	29
Dietary Supplement - Multivitamin	29
Local Anesthetic / Pain Management	29
Systemic Antibiotic - Macrolides	28
ADHD Non-Stimulant - Norepinephrine Reuptake Inhibitor	26
Hematologic Agents	24
Inhaled Corticosteroid	24
Miscellaneous Neurological Treatments	24
Topical Treatments - Miscellaneous	24
Dietary Supplement - Vitamin C (Ascorbic Acid)	23
Respiratory - Therapeutic Support	22
Gabapentinoid (for Neuropathic Pain and Seizure Disorders)	21
Endocrine Medication - Antidiuretic Hormone Analog	19
Autoimmune / Inflammatory Disease	18
Dietary Supplement - Vitamin A	18
Digestive Health - Enzyme Supplement	17
Systemic Antibiotic - Sulfonamides and TMP	17
Treatments of Rare Genetic Diseases	17
Hormonal Modulator - Anti-Androgen / Acne Treatment	16
Dietary Supplement - Vitamin A and Vitamin D	15
Growth Hormone Therapy	14
Movement Disorder Treatments	14
Neurological - seizure/migraine	14
Dietary Supplement - Vitamin E	13
Emergency/Supportive Medications	11
Substance Use Disorder Treatments	11
Topical Antihistamines	11
Dietary Supplement - Vitamin K	9
Immune and Allergy Support	9
Specialty Neurological and Autoimmune	9
PTSD Treatment - Alpha Blocker	8
General Anesthetic	7
Bile Acid Therapy / Gallstone Management	6
Caffeine-Based Stimulant	6
Dietary Supplement - Zinc	6
Gout Treatment - Anti-inflammatory	6
Cannabinoid Medication - Other	5

medRxiv preprint doi: https://doi.org/10.1101/2025.11.19.25340321; this version posted November 20, 2025. The copyright holder for this preprint (which was not certified by peer review) is the author/funder, who has granted medRxiv a license to display the preprint in perpetuity.

It is made available under a CC-BY 4.0 International license .

Miscellaneous Hormone Therapy	5
Dietary Supplement - Vitamin A and Vitamin E	4
